# Comparative gene expression analysis of planktonic *Porphyromonas gingivalis* ATCC 33277 in the presence of a growing biofilm *versus* planktonic cells

**DOI:** 10.1186/s12866-019-1423-9

**Published:** 2019-03-12

**Authors:** María C. Sánchez, Patricia Romero-Lastra, Honorato Ribeiro-Vidal, Arancha Llama-Palacios, Elena Figuero, David Herrera, Mariano Sanz

**Affiliations:** 1 Laboratory of Dental Research, University Complutense, Madrid, Spain; 2 Etiology and Therapy of Periodontal Diseases (ETEP) Research Group, University Complutense, Madrid, Spain; 3 Department of Dental Clinical Specialities (DDCS), Faculty of Odontology, Plaza Ramón y Cajal s/n Ciudad Universitaria, 28040 Madrid, Spain

**Keywords:** *Porphyromonas gingivalis*, Microarray hybridization, Gene transcription, Transcriptomics, Gene expression, RT-qPCR, Planktonic growth, Biofilm

## Abstract

**Background:**

*Porphyromonas gingivalis*, a microorganism residing in the oral cavity within complex multispecies biofilms, is one of the keystone pathogens in the onset and progression of periodontitis. In this in vitro study, using DNA microarray, we investigate the differential gene expression of *Porphyromonas gingivalis* ATCC 33277 when growing in the presence or in absence of its own monospecies biofilm.

**Results:**

Approximately 1.5% of genes (28 out of 1909 genes, at 1.5 fold change or more, *p*-value < 0.05) were differentially expressed by *P. gingivalis* cells when in the presence of a biofilm. These genes were predominantly related to the metabolism of iron, bacterial adhesion, invasion, virulence and quorum-sensing system. The results from microarrays were consistent with those obtained by RT-qPCR.

**Conclusion:**

This study provides insight on the transcriptional changes of planktonic *P. gingivalis* cells when growing in the presence of a biofilm. The resulting phenotypes provide information on changes occurring in the gene expression of this pathogen.

## Background

*Porphyromonas gingivalis*, a Gram-negative anaerobe residing within the oral cavity, has been identified as one of the key pathogenic species implicated in the establishment and development of periodontal diseases [1–3], mainly through the expression of a broad range of virulence factors involved in tissue colonization, evasion of host defenses and stimulation of a chronic inflammatory response [3].

*P. gingivalis* is found within the oral cavity adopting a sessile biofilm lifestyle, predominately as a component of complex biofilms containing multiple microbial communities [4]. *P. gingivalis* has the capacity to adhere to mucosal and dental surfaces, including the teeth, gingiva, cheek and tongue, as well as to other oral bacteria, thus withstanding the host natural barriers, the host immune defenses and the presence of multiple antimicrobial agents [5–8]. These virulence factors are the consequence of specific gene expression, and it is, therefore, important to improve our knowledge on them in order to understand how these microorganisms can adapt to specific ecological determinants, and their role within the biofilm environment.

It is well known that microbial gene expression is significantly different within biofilms when compared to planktonic growth [9–12]. Recent investigations have studied the specific gene expression of *P. gingivalis* when growing in biofilms, and compared to planktonic growth, demonstrating the differential expression of a broad range of genes [13–19]. In biofilm growth, the cell replication and growth rate are decreased with repressed genes involved in cell envelope biogenesis, DNA replication, energy production, biosynthesis and phospholipid metabolism. By contrast, there is an important number of genes involved in regulatory mechanisms which are overexpressed, such as those encoding transport and binding proteins, proteins involved in signal transduction and transcriptional regulation and many others not well characterized. Within this research line, Ang et al. (2008) [20] conducted a proteomic study comparing the envelope proteins of *P. gingivalis*, either growing in planktonic or in biofilms, demonstrating an overexpression of proteins involved in hemin transport (HmuY and IhtB), in metabolic pathways, virulence factors or proteins of the cell envelope.

Other investigations have studied the overexpression of *P. gingivalis* genes affecting structural characteristics such as the presence of different fimbrial types, as well as specific polysaccharides involved in adhesion and colonization mechanisms, which may significantly contribute to biofilm formation [21–23]. Also, cell-cell signaling mechanisms has been the focus of study, and *P. gingivalis* cells have shown up-regulation of the LuxS-dependent signaling regulatory genes, involved in inter-species communication and quorum sensing but also involved in stress, protease modulation and haemagglutination or in hemin uptake [24–27].

In spite of this knowledge, the genetic and environmental determinants affecting *P. gingivalis* cells when transiting from free-floating cells to biofilm have not been fully elucidated [28–30]. It was, therefore, the purpose of this investigation to study the gene expression of free-floating *P. gingivalis* cells either growing in a pure planktonic environment or when they are placed in the presence of a *P. gingivalis* mono-species biofilm.

## Material and methods

### Bacterial strain and culture conditions

*Porphyromonas gingivalis* ATCC 33277 was used in this study and grown on supplemented blood agar plates [Blood Agar Oxoid No 2; Oxoid, Basingstoke, UK; with 5.0 mg/L hemin (Sigma, St. Louis, MO, USA), 1.0 mg/L menadione (Merck, Darmstadt, Germany) and 5% (*v*/v) sterile horse blood (Oxoid)], at 37 °C for 48 h in anaerobiosis (10% H_2_, 10% CO_2_, and 80% N_2_).

### Experimental assays

**Fig. 1:**
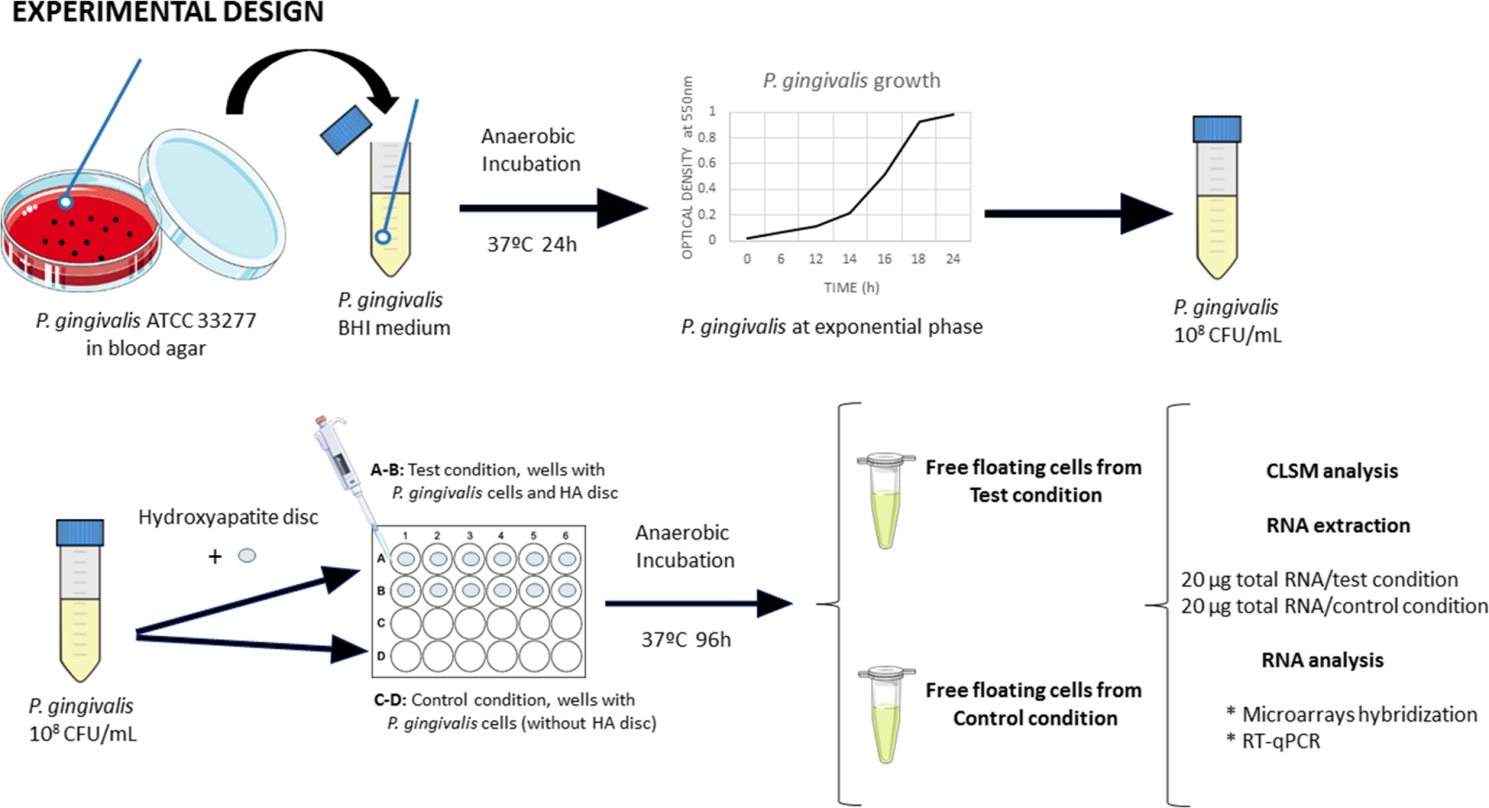
Overview of experimental design. *Porphyromonas gingivalis* ATCC 33277 strain was maintained on blood agar plates and grown in modified Brain Heart Infusion (BHI) medium. After 24 h, optical density was measured, and a pure culture containing 10^8^ colony forming units per milliliter (CFU/mL) was set. Two culture conditions were then prepared: Test cells, depositing *P. gingivalis* cells in presence of Hydroxyapatite (HA) disc, and Control cells, depositing *P. gingivalis* cells in the wells without HA discs. After 96 h of incubation of multi-well plates under anaerobic conditions, free floating *P. gingivalis* cells from both test and control condition were harvested, examined by CLSM, processed and total RNA extracted and purified. Agilent Oligo Microarrays 8x15K (074976) for *P. gingivalis* ATCC 33277 were used for hybridizations, (this slides also contents probes against the whole genome of *P. gingivalis* W83), and RT-qPCR analyses were performed to confirm the results. The experiments were repeated three times and each experimental condition was pooled into the three biological replicates and processed for the transcriptomic analysis. (Images for Fig. 1 were taken from https://smart.servier.com/ under a creative commons licence)

Figure 1 depicts an overview of the experimental design*. P. gingivalis* ATCC 33277 was grown in modified Brain Heart Infusion (BHI) medium (Becton, Dickinson and Company, USA) in anaerobiosis at 37 °C for 24 h [13]. Bacteria were harvested in their late exponential growth phase [0.8; standard deviation (SD) =0.1 of optical density at 550 nm] and added to fresh modified BHI medium in order to obtain a pure culture containing 10^8^ colony-forming units per milliliter (CFU/mL). Two experimental groups were carried out, free-floating *P. gingivalis* cells either growing in a pure planktonic environment or when they are placed in the presence of a *P. gingivalis* mono-species biofilm. For that, the planktonic bacterial culture prepared was placed in pre-sterilized polystyrene well tissue culture plates (Greiner Bio-one, Frickenhausen, Germany) under two environmental conditions:

1. Test group: the bacterial suspension of *P. gingivalis* was deposited in the culture plates containing sterile ceramic calcium hydroxyapatite (HA) discs [7-mm in diameter and 1.8 mm in thickness (SD = 0.2); (Clarkson Chromatography Products, Williamsport, PA, USA)].
2. Control group: the bacterial suspension of *P. gingivalis* was deposited in the plates without HA discs.

Plates were incubated in anaerobic conditions at 37 °C for 96 h. Wells containing only culture medium were also evaluated to assure sterility and lack of contamination.

After 96 h of incubation, free-floating *P. gingivalis* cells from both groups were harvested and processed for the transcriptomic analysis, with the goal of obtaining approximately 20 μg of total RNA for each replicate. Each pooled sample corresponded to one biological replicate and was processed in the same manner.

### Confocal laser scanning microscopy (CLSM)

Test and control group samples were analyzed by confocal laser scanning microscopy (CLSM) after 96 h of growth. 100 μL of free-floating *P. gingivalis* cells from both test and control groups were deposited in a slide and were stained with the fluorochrome Syto9 (wave lengths 515–530 nm) for 9 min at room temperature to obtain an optimum fluorescence signal (Molecular Probes B. V., Leiden, The Netherlands). Samples were then covered with a coverslip, with the aim of minimizing the movement of bacteria. CLSM clearly showed the absence of sessile phenotype in the bottom of the plate of control group samples, while a sessile phenotype was developed on the surface of hydroxyapatite discs of test group. For that, after completely removing the free-floating *P. gingivalis* cells from control wells, these ones were sequentially rinsed with 2 mL of sterile PBS three times (time per rinse, 10 s), in order to remove unbound bacteria. In the same way, after completely removing the free-floating *P. gingivalis* cells from test wells, HA discs from test group were rinsed by immersion in 2 mL of sterile Phosphate-Buffered Saline (PBS) three times (time per rinse, 10 s). Then, the bottom of the plate and the HA discs were stained with the fluorochrome for 9 min at room temperature. Samples were observed with CLSM [Ix83 Olympus® inverted microscope coupled to an Olympus® FV1200 Confocal System (Olympus; Shinjuku, Tokyo, Japan) using × 63 water-immersion lenses (Olympus) The CLSM control software was set to take a z-series of scans (xyz) of 0.5 μm thickness (8 bits, 1024 × 1024 pixels). Image stacks were analyzed with the Olympus® software (Olympus).

### Total RNA isolation

After 96 h of incubation under anaerobic conditions, free-floating *P. gingivalis* cells from both test and control groups were harvested, and total RNA was isolated using the TRIzol® Max Bacterial RNA Isolation Kit (Ambion, Life Technologies, Carlsbad, CA, USA) as detail in Romero-Lastra et al. (2017) [13]. Briefly, *P. gingivalis* cell pellets from both groups were resuspended in Max Bacterial Reagent® (Ambion) and after temperature shock treatment to help the break of the cell wall, consisting on 4 min incubation at 95 °C and cooling on ice for 10 min, TRIzol® reagent (Ambion) was added. Total RNA was extracted using the chloroform protocol and isopropanol precipitation. Isolated RNA was then washed in 75% ethanol and resuspended in 50 μl RNase-free water (Water PCR grade, Roche Diagnostic GmbH; Mannheim, Germany). To remove any contaminating DNA, samples were then treated with DNase I (Ambion), and purified using columns of RNeasy Mini kit (Qiagen) according to the manufacturer’s protocol.

RNA concentration was measured by NanoDrop ND1000 spectrophotometer (NanoDropTechnologies; Thermo Scientific™, LLC, Wilmington, DE, USA), and RNA integrity was assessed using an automated electrophoresis device (Agilent 2100 Bioanalyzer, Agilent Technologies, Santa Clara, CA, USA). An A260/A280 ratio of at least 2.0 was considered appropriate for the experiments.

### cDNA synthesis and transcriptomic protocol

All experiments were done in triplicates. The fluorescently labeling was performed using SuperScript Indirect cDNA Labeling System (Invitrogen; Carlsbad, CA, USA) as described in Romero-Lastra et al. (2017) [13]. Preparation of probes and hybridization was performed as described in the manufacturer’s instructions [One-Color Microarray Based Gene Expression Analysis Manual Ver. 6.5 (Agilent Technologies)].

Slides specific for the strain *P. gingivalis* ATCC 33277 [Agilent Oligo Microarrays 8x15K (074976)] were used. The array also contents the whole genome of *P. gingivalis* W83.

### Microarray and data analysis

Images from Cy3 one-color microarrays (Agilent) were taken, corrected and analyzed following the protocol detailed in Romero-Lastra et al. (2017) [13].

LIMMA language with “normexp” and loess methods were used to treat background correction and normalization [31, 32]. Log-ratio values were used for consistency among arrays [31]. Differentially expressed genes were determined using linear models and Bayes moderated t-statistic; Benjamani and Hochberg method was used to correct false discovery rate *p*-values [31, 32], and was controlled to be lower than 5% and a cutoff of fold change (increase or decrease) up to 1.5 between the two situations. Expression ratios were expressed as means of the fold changes of the three biological replicates and Standard Deviation (SD). Hybridizations and statistical analysis were performed by the Genomics Unit at the National Center of Biotechnology at the Universidad Autónoma of Madrid (Spain).

### Confirmation of microarray data by reverse transcription-quantitative polymerase chain reaction (RT-qPCR)

Microarray results were confirmed by RT-qPCR selecting 8 genes differentially expressed, four genes from the up-regulated group and four from the down-regulated one. Universal Probe Library Roche software tool (Roche Diagnostics) was used to design specific primers (Table 1). *P. gingivalis* 16S rRNA gene was used as a loading control.

**Table 1:** Primers used for reverse transcription quantitative polymerase chain reaction (RT-qPCR)

Locus name	Putative identification	Primers for RT-qPCR	
PGN_0557 (*hmuR)*	TonB-dependent receptor HmuR	Forward Backward	5′-3′: TAGTCGCGACGGACAGAAAT 5′-3′: CTGGTGAAGATCCCACGTTT
PGN_1058 (*ftn)*	Ferritin	Forward Backward	5′-3′: GAAATGATCGAGGCTGTCGT 5′-3′: GTCCTGTGATGCCATATCTCC
PGN_0780 *(prtQ)*	PrtQ, protease	Forward Backward	5′-3′: CAGCTGTAAACCGCAACAAG 5′-3′: GGCTTGGCTCCCGTATTATC
PG_0437	Polysaccharide biosynthesis/export protein	Forward Backward	5′-3′: AGAGGGCCTTACTCGTACCG 5′-3′:CCACTGGAAATAATCCTCTTCTGT
PGN_0183 *(fimC)*	Minor component FimC	Forward Backward	5′-3′:CCTTTTCAAGAAAGAACTTGAGGA 5′-3′: GTCGGACTATCGGCTCGTT
PG_2131	OmpA_c-like	Forward Backward	5′-3′: ACACACCCCTCTCGTCTGAG 5′-3′: TCCCTTCCGGATAGCTCTG
PGN_0181	Fimbrillin-A associated anchor proteins Mfa1 and Mfa2	Forward Backward	5′-3′: CCACTACGGTGTCTTTCGTG 5′-3′: TTAGACGCTTTGCACATTGG
PG_1712	Alpha-1,2-mannosidase family protein	Forward Backward	5′-3′: GCTACGAAAGCCGTCCATC 5′-3′: GTACCACTCCCAACCTTTGC

RT-qPCR was performed from the cDNA generated from 1 μg of total RNA from each sample, using the High Capacity cDNA Archive Kit (Applied Biosystems, ThermoFisher Scientific; MA, USA) in a final reaction volume of 10 μL. The qPCR reactions were performed in triplicate using 5 μL per well of each cDNA, and 3 μL of a mix composed by 0.4 μM of each primer, 5x HOT FIREPol1 EvaGreen1 qPCR Mix Plus (ROX), and nuclease-free water, to reach a final volume of 8 μL in 384-well optical plates and following the standards protocols provided by ThermoFisher Scientific in an Applied Biosystems ABI PRISM 7900HT apparatus (95 °C 10 min, 40 cycles of 95 °C 15 s and 60 °C 60 s, and a final standard melting curve dissociation protocol). The results of differentially expressed genes were analysed using the Expression Suite Software Version 1.1 and Comparative Ct Method (ΔΔCt) was applied [33].

## Results

**Fig. 2:**
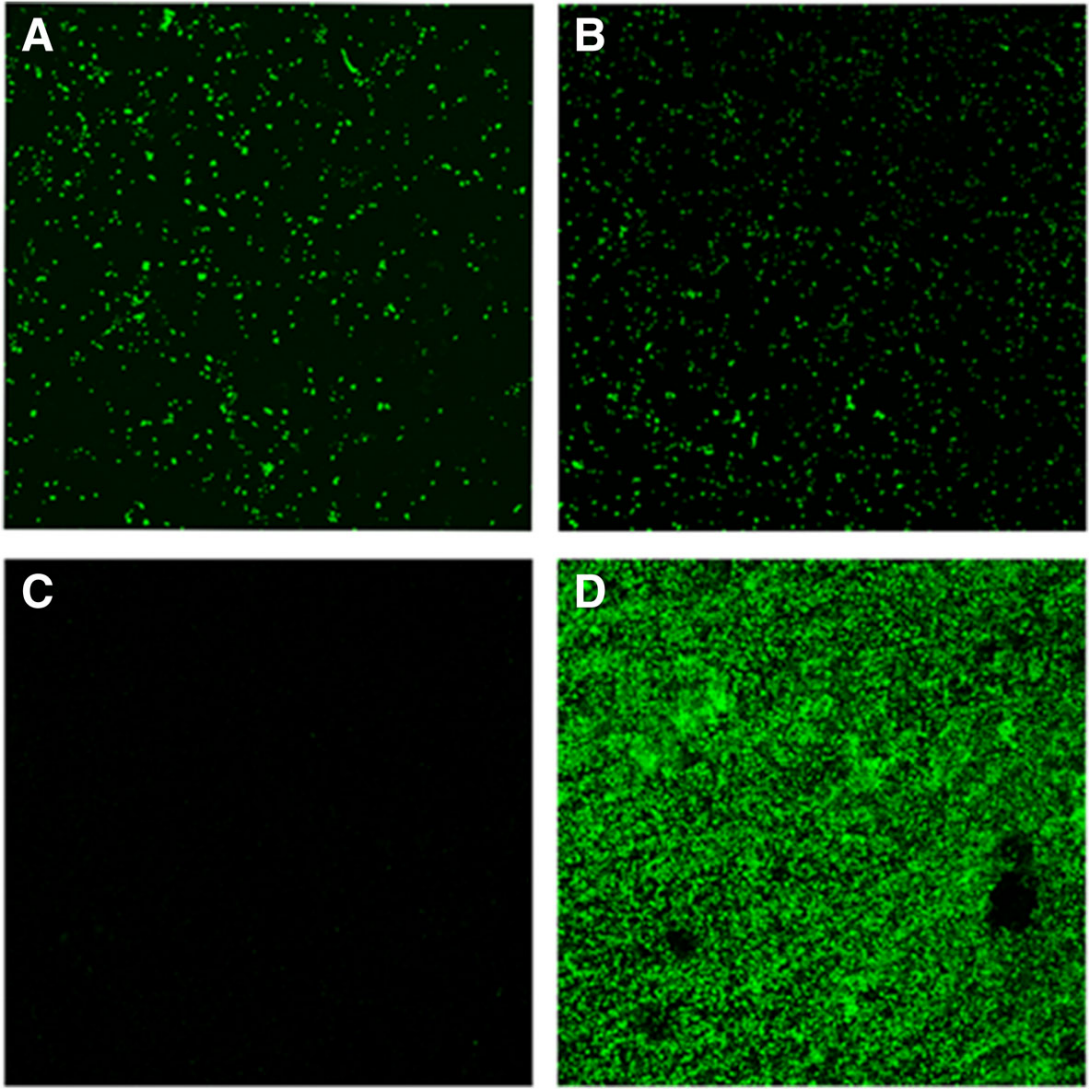
Representative confocal micrographs of *P. gingivalis* depicting 2D maximum projection images after 96 h of incubation of the two experimental groups designed, (**a**) planktonic *P. gingivalis* cells growing in a pure planktonic environment and (**b**) free-floating *P. gingivalis* cells placed in the presence of a *P. gingivalis* mono-species biofilm. Image (**c**) corroborate the absence of sessile phenotype on the bottom of the plate of control group samples, only faint debris could be observed adhered. Image (**d**) shows a sessile phenotype evolved on the surface of hydroxyapatite discs form the test group. Specimens were stained with Syto9 fluorochrome (Molecular Probes B. V., Leiden, The Netherlands)

By CLSM analysis it was verified that in the control group, after 96 h of incubation, free-floating *P. gingivalis* cells had not evolved to biofilm phenotype (Fig. 2 a). It could be observed that no sessile phenotype was formed on the bottom of the plate, observing only some cellular debris (Fig. 2 c). In contrast, in the test samples, free-floating *P. gingivalis* cells (Fig. 2 b) were in presence of biofilm evolved on HA disc (Fig. 2d).

**Fig. 3:**
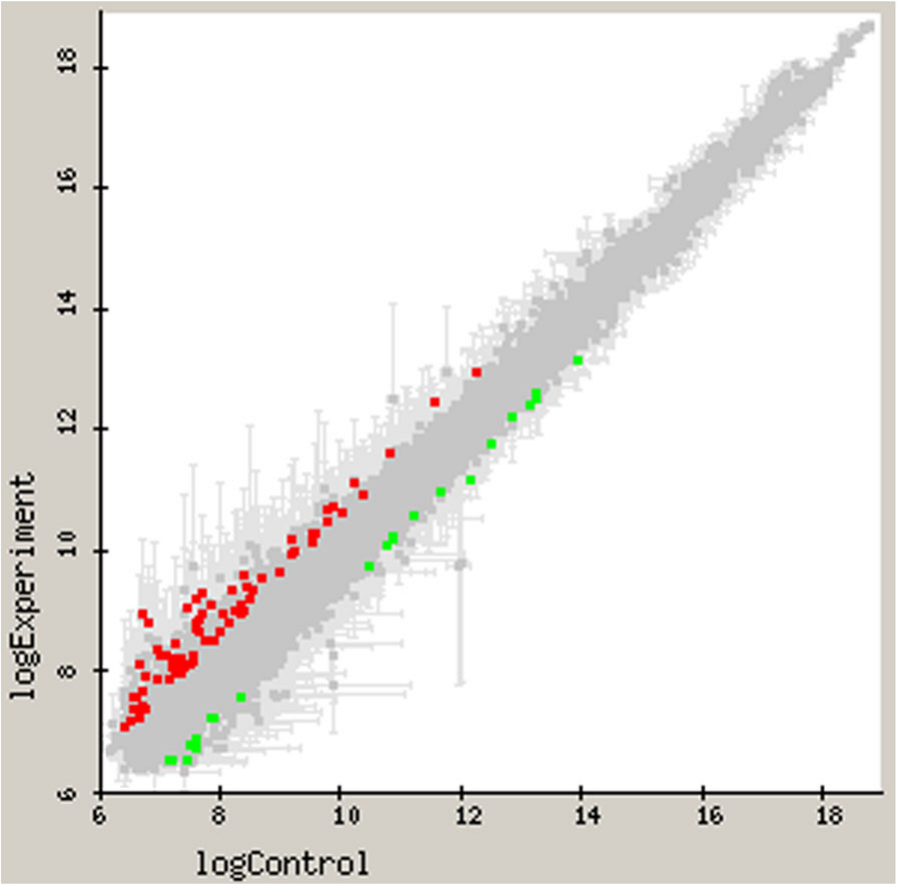
Differential gene expression by comparative microarray analyses (represented in log_10_) when comparing planktonic *Porphyromonas gingivalis* ATCC 33277 cells either in presence of a growing biofilm or in absence of a biofilm. Control planktonic cell gene expression (X-axis) is plotted against test cells (Y-axis) with a 1.5 fold change (up or down) and *p*-value < 0.05. Down regulated genes (green) and up-regulated genes (red) are shown in the figure

The Fig. 3 depicts the genes with differential expression in *P. gingivalis* ATCC 33277 resulting from microarray-based transcriptome analyses, when comparing the two planktonic states, either in presence or absence of a bacterial biofilm. Differentially expressed genes with 1.5 fold change (up or down) and *p*-value < 0.05 were plotted, X-axis represents log_10_ expression of pure planktonic state (in absence of a biofilm) and Y-axis shows the log_10_ expression genes of cells in the presence a growing biofilm.

The differentially regulated genes of *P. gingivalis* cells under both test and control groups are depicted in Table 2. Expression ratios were expressed as means of the fold changes of the three biological replicates and Standard Deviation (SD). Although the gene expression was not markedly different between both groups, a total of 28 out of 1909 genes (1.5%) were differentially expressed by free-floating *P. gingivalis* cells growing in the presence of a biofilm.

**Table 2:** Differentially expressed genes in free-floating *Porphyromonas gingivalis* ATCC 33277 cells either in presence of a growing biofilm or in pure planktonic growth (cutoff ratio ± 1.5-fold change, p-value < 0.05) for the microarray analysis

Open reading frame^a^	Gene^a^	Protein or function	Expression ratio by Microarray^b^ (SD)
PGN_0181		Fimbrillin-A associated anchor proteins Mfa1 and Mfa2	−1.60 (0.09)
PGN_0183	*fimC*	Minor component FimC	−1.76 (0.16)
PGN_0493		Heavy-metal-associated domain (Hma)	+ 1.56 (0.03)
PGN_0495		Conserved hypothetical protein	+ 1.73 (0.15)
PGN_0529	*batA*	Aerotolerance-related membrane protein BatA	−1.62 (0.02)
PGN_0557	*hmuR*	TonB-dependent receptor HmuR	+ 1.67 (0.13)
PGN_0604		Ferritin	+ 1.78 (0.08)
PGN_0649		Conserved hypothetical protein	+ 1.78 (0.18)
PGN_0780	*prtQ*	PrtQ, protease	+ 1.84 (0.41)
PGN_0787		Conserved hypothetical protein	+ 1.61 (0.06)
PGN_1058	*ftn*	Ferritin	+ 1.74 (0.12)
PGN_1093		Conserved hypothetical protein	−1.61 (0.14)
PGN_1206	*folD*	Methylenetetrahydrofolate dehydrogenase/ cyclohydrolase	+ 1.86 (0.25)
PGN_1312		Probable transcriptional regulator as Arg-repressor	+ 1.85 (0.47)
PGN_1494		Putative oxygen-independent coproporphyrinogen III	+ 2.02 (0.02)
PGN_1534		Hypothetical protein	+ 1.95 (0.28)
PGN_2071	*topA*	DNA topoisomerase I	+ 2.26 (0.18)
PG_0009		ISPg5 transposase Orf1	+ 1.74 (0.16)
PG_0437		Polysaccharide export protein, BexD/CtrA/VexA family	+ 1.89 (0.42)
PG_0718		Conserved hypothetical protein	+ 1.99 (0.38)
PG_0942		ISPg5 transposase Orf1	+ 1.59 (0.01)
PG_1169		Hypothetical protein	+ 1.93 (0.28)
PG_1403		Rhomboid family protein	+ 1.70 (0.09)
PG_1712		Alpha-1,2-mannosidase family protein	−1.52 (0.02)
PG_1979		Hypothetical protein	+ 1.82 (0.26)
PG_2094		Conserved domain protein	+ 1.91 (0.32)
PG_2130		Hypothetical protein	−1.58 (0.09)
PG_2131		OmpA_C-like	−1.61 (0.04)

^a^Putative identification from Genebank. ^b^ Results of three biological replicates. Expression ratio by Microarray indicates the mean fold expression (SD) of that gene

From these differentially expressed genes, 21 transcripts were found significantly increased (Table 2). These included genes related to iron acquisition and storage, among them the PGN_0557 (*hmuR)* gene, which encodes for the outer-membrane hemin utilization receptor involved in the uptake of both free hemin and heme bound to hemoproteins. Also, the gene PGN_0493 appeared overexpressed, which encodes for a receptor Hma, implicated in heme uptake. Similarly, the PGN_1494 gene, which encodes for the oxygen-independent coproporphyrinogen-III oxidase, or the genes PGN_1058 (*ftn)* and PGN_0604*,* which are related with intracellular iron-storage proteins, were differentially expressed. *P. gingivalis*, under these test conditions overexpressed genes encoding for transposases (PG_0009 and PG_0942), in particular the ISPg5 transposase Orf1, and the gene PGN_0780 *(prtQ)*, which encodes a protease with peptidase activity, belonging to the PrtC family of genes encoding collagenase-like proteases. The gene PGN_1312, which encodes a transcriptional regulator of arginine metabolism and the gene PG_0437 involved in the biosynthesis of polysaccharides, was also up-regulated.

Conversely, in the test conditions seven transcripts demonstrated significant down-regulation, being these genes related to adhesion or virulence (PGN_0183 (*fimC)*, PGN_0181, PG_2130, PG_2131, PG_1712) (Table 2).

**Fig. 4:**
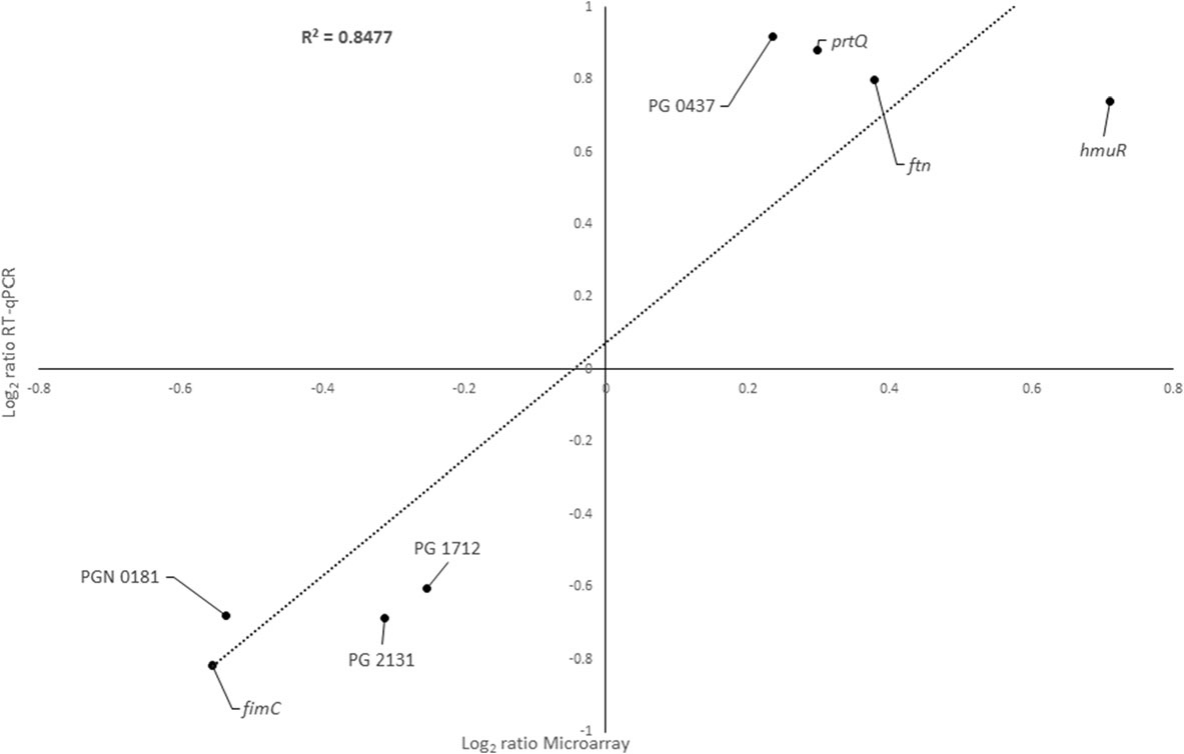
Graph depicting the correlation between the log_2_ ratio Microarray and Reverse Transcription-quantitative Polymerase Chain Reaction (RT-qPCR) gene expression ratios when test and control conditions were compared. The RT-qPCR log_2_ values were plotted against the microarray data log_2_ values (R^2^ = 0.8477)

RT-qPCR was used for the assessment of the microarray results from the selected genes (4 from the up-regulated and 4 from the down-regulated group). Figure 4 shows a high correlation between the log_2_ ratio of microarray and RT-qPCR results in the two studied conditions (R^2^ = 0.8477).

## Discussion

This in vitro investigation has shown that a 1.5% of genes (28 out of 1909 genes, at 1.5-fold change or more, *p*-value < 0.05) were differentially expressed by *P. gingivalis* cells when in the presence of a biofilm. Several studies have investigated the differential gene expression of the periodontal pathogen *P. gingivalis* when growing under different conditions, basically under planktonic or biofilm conditions, and have reported distinct genetic expression [13–19]. Among them, our research group reported differences in gene expression when *P. gingivalis* ATCC 33277 grew either in planktonic or in biofilms states, finding that 4.8% of genes were differentially expressed when growing in biofilm. These up-regulated genes were mainly related to the cell envelope, transport, and binding or outer membranes proteins, while the down-regulated genes were mainly genes related to transposases or oxidative stress [13]. Most of the previous studies, however, have not elucidated the differential gene expression when this bacterium is in transition between both states. That is, whether planktonic *P. gingivalis* within an environment of biofilm growth, may undertake gene expression changes that would facilitate its adaptation to the developing biofilm environment.

The present study has tried to reproduce experimentally the situation in which free-floating *P. gingivalis* cells when in the presence of a *P. gingivalis* mono-species biofilm will develop a differential genetic expression when compared with similar planktonic *P. gingivalis* cells but without any biofilm influence. To define differential expression, apart from statistical significance (*p* < 0.05), a threshold of 1.5-fold (up or down) in the average expression ratio was selected, which has been previously used in microarray analysis of gene expression in *P. gingivalis* and was considered biologically relevant [14, 18, 26]. Under these experimental conditions, a total of 28 out of 1909 genes (1.5%) of *P. gingivalis* were differentially expressed when cells from the test group were compared with the control.

Among the differentially expressed genes, the gene PGN_0557 (*hmuR)* showed up-regulation [+ 1.67 (SD = 0.13)], which encodes a major hemin uptake protein, but also a potential adhesin. The role of this adhesin PGN_0557 (*hmuR*) has been demonstrated by Kuboniwa et al. (2009) [17], using an in vitro biofilm model of three bacterial species (*F. nucleatum* subsp. *nucleatum*, *P. gingivalis* ATCC 33277 and *Streptococcus gordonii* DL1), demonstrating that the lack of PGN_0557 (*hmuR)* gene in *P. gingivalis* resulted in a 70% reduction of community formation. In addition, the four genes involved in iron transport (PGN_1058 (*ftn)*, PGN_0604, PGN_1494 and PGN_0493) appeared up-regulated, what may indicate that these genes may contribute to biofilm formation by protecting *P. gingivalis* from oxidative stresses generated by intracellular free iron. One of these genes, PGN_0493, encodes for the receptor Hma, which has also been implicated in heme uptake. Hagan and Mobley (2009) [34] demonstrated that iron acquisition, mediated by specific outer membrane receptors, was critical for the colonization of the urinary tract by *Escherichia coli*. Similarly, heme acquisition facilitated by the receptor Hma was a pre-requisite for the development of kidney infection by *E. coli*. Therefore, the results obtained may indicate that *P. gingivalis* would use similar colonization mechanisms to develop biofilm state.

Under these experimental conditions, the gene PG_2094 was up-regulated [+ 1.91 (SD = 0.32)]. It is classified as a hypothetical protein but with certain homology with the LuxR family transcriptional regulator of *P. gingivalis* SJD2, involved in the quorum sensing system in bacteria [35]. This might suggest a promotion of cell communication in *P. gingivalis* when these cells are in the presence of a biofilm. However, nowadays, although the homology with this domain is preserved, a plausible function was assigned: translocation/assembly module TamB. Bacteria export proteins across the cell envelope using diverse systems. The secretion mechanisms fulfill general cellular functions but are also essential for pathogenic bacteria during the interaction with the host cells. Selkrig et al. (2012) described a new translocation and assembly module (TAM) that promotes efficient secretion of autotransporters in proteobacteria. Functional analysis of the TAM in several bacteria, *Salmonella enterica, Citrobacter rodentium* or *E. coli* demonstrated that TamB is an integral inner membrane protein that forms the translocation and assembly module or TAM complex [36] with the outer membrane protein, TamA, an Omp85-family protein. The discovery of the TAM provides a new target for the development of therapies to inhibit colonization by bacterial pathogens [37].

It must be noted that, involved in inter-species communication and quorum sensing, LuxS-dependent signaling regulatory genes have been also related to stress, protease modulation and haemagglutination or in hemin uptake [24–27, 38]. Studies using LuxS-deficient mutants of *P. gingivalis* have reported an altered expression of genes involved in hemin uptake, specifically up-regulation of the genes for a TonB-linked hemin binding protein, HmuR and the iron storage protein ferritin [27, 39, 40]. In the present study, and under these experimental conditions, genes related with the synthesis of both proteins were up-regulated [PGN_0557 (*hmuR)* [+ 1.67 (SD = 0.13)], PGN_0604 [+ 1.78 (SD = 0.08)] and PGN_1058 (*ftn)* [+ 1.74 (SD = 0.12)]. In low level conditions of hemin and iron, HmuR production is suppressed by LuxS signaling, and thus the requirement of ferritin for iron storage should be reduced [40, 41]. Instead, when *P. gingivalis* has availability of heme/iron, as occurred in the conditions used in vitro with 5.0 mg/L hemin concentration or in vivo when the established bacterial community starts to destroy the periodontal tissue, reduced Autoinducer-2 (AI-2) expression, removes the repression of LuxS over *hmuR* gene, and subsequently of ferritin, which facilitate tight control and ensure adaptability to environmental conditions [40–42].

Free-floating *P. gingivalis* cells in the presence of a biofilm also overexpressed the genes PG_0009 and PG_0942, which encode for transposases, in particular the insertion sequence (IS) elements and ISPg5 transposase Orf1. These results are in agreement with those reported by Califano et al. (2000) [43], that described how ISPg5 and others IS elements could contribute to the diversity of *P. gingivalis* strains, as a mode of adapting to specific ecological determinants. This differential regulation in transposases genes and transposon functions has also been reported by our research group [13], in the comparative gene expression analysis of *P. gingivalis* ATCC 33277 in biofilm versus planktonic cells, and has been attributed to an adaptation to the changes to the new phenotypic state. The results from this current investigation could indicate that *P. gingivalis* begins to adapt to different environmental conditions, and may gradually adopt a sessile phenotype growth.

Among other genes related to a biofilm formation, PG_0437 showed + 1.89 fold changed expression (SD = 0.42). This gene encodes a polysaccharide outer membrane protein exporter, which is involved in polysaccharide biosynthesis. Haft et al. (2006) reported that the overexpression of these proteins occurred preferentially in bacteria from sediments, soils and biofilms [44], so that these results may imply that, under the experimental conditions used, *P. gingivalis* cells would use this system when adapting to evolve to a biofilm state.

The genes PGN_1312 and PGN_0780 *(prtQ)* encoding proteases were also up-regulated in the test group. *P. gingivalis* cells in the presence and, possibly evolving into a biofilm, may develop peptidase activity, with the purpose of inactivating host defense mechanisms, what may be relevant when this pathogen is forming a biofilm [45, 46].

Conversely, several genes encoding proteins involved in bacterial adhesion, invasion or virulence, were identified as down-regulated in free-floating *P. gingivalis* cells in the presence of a biofilm (PGN_0183 (*fimC)*, PGN_0181, PG_2130, PG_2131, PG_1712). In fact, *P. gingivalis* fimbriae have been identified as one of its major colonization factors [47–53]. Although these results demonstrating the down-regulation of the genes encoding for the formation of fimbriae may appear contradictory, these genes encode only minor components of the fimbriae proteins FimA and Mfa1. In the comparative gene expression analysis of *P. gingivalis* ATCC 33277, in planktonic versus mature biofilms states, carried out by our research group, it was reported that *fimD*, one of the minor components of the fimbriae A, appeared down-regulated in biofilm state [13]. Similarly, Krogfelt and Klemm (1988) [54] showed that a clone of *E. coli* lacking the genes encoding these minor component proteins did produce fimbriae consisting of pure Fim A protein, indicating that these minor protein components were not necessary for the structural integrity of the fimbriae. These results are also coincident with those reported by Nagano et al. (2012), which demonstrated that despite a lack of fimC, the FimA protein was still produced and polymerized to form fimbriae [53]; likewise Nishiyama et al. (2007) reported that the complete deletion of PGN_0181 did not affect the formation of FimA fimbriae by *P. gingivalis* ATCC 33277 [55]. Similarly, previous works demonstrated that *P. gingivalis* autoaggregation, and its subsequent biofilm initiation, are probably due to FimA [56, 57], this autoaggregation is intensified by a loss of short fimbriae, however, other authors claim that Mfa fimbriae is essential in the process of colony formation on solid surfaces [57, 58]. These reports appear to indicate the assay- and context-dependency in assessing the role of each fimbrial type.

PG_1712 gene encoding the protein alpha-1,2-mannosidase was also down-regulated in test group. This protein belongs to the glycosidic hydrolase family 92, cleaving mannose, which is an important sugar in the synthesis of glycoproteins by Gram-negative bacterium as *P. gingivalis*. Five genes hydrolyzing mannan have been reported in *P. gingivalis* (PG_0032, PG_0902, PG_0973, PG_1711, and PG_1712), although the resulting enzymes have not been well characterized, thus making it difficult to interpretate these results. Rangarajan et al. (2013) reported that α- and β-mannosidases from *P. gingivalis* did not have an effect on the biosynthesis of O-LPS and A-LPS or in the secretion of Arg-gingipains [59].

## Conclusions

This in vitro investigation has demonstrated that 28 genes (1.5%) were differentially expressed (up-regulated or down-regulated) when comparing free-floating *P. gingivalis* placed in the presence of a *P. gingivalis* mono-species biofilm versus cells growing in a pure planktonic environment. Most of these genes are related to the metabolism of iron, bacterial adhesion, invasion, virulence and quorum-sensing system. Although the differential gene expression between *P*. *gingivalis* planktonic cells growing under the test or control conditions may seem limited, the thorough understanding of the genes and its regulatory pathways involved in the transition between planktonic and biofilm states may provide important insights in the prevention of biofilm formation and consequently of periodontal diseases.

## Abbreviations

BHI: Brain Heart Infusion culture medium
CFU/mL: Colony-forming units per milliliter
CLSM: Confocal Laser Scanning Microscopy
DNA: Deoxyribonucleic acid
HA: Hydroxyapatite
RT-qPCR: Reverse Transcription Quantitative Polymerase Chain Reaction
SD: Standard deviation
